# Targeting Hepatic Stellate Cells for the Treatment of Liver Fibrosis by Natural Products: Is It the Dawning of a New Era?

**DOI:** 10.3389/fphar.2020.00548

**Published:** 2020-04-30

**Authors:** Yau-Tuen Chan, Ning Wang, Hor Yue Tan, Sha Li, Yibin Feng

**Affiliations:** School of Chinese Medicine, The University of Hong Kong, Hong Kong, Hong Kong

**Keywords:** hepatic stellate cells, liver fibrosis, cirrhosis, natural product, herbal medicine, network pharmacology

## Abstract

Liver fibrosis is a progressive liver damage condition that is worth studying widely. It is important to target and alleviate the disease at an early stage before turning into later cirrhosis or liver cancer. There are currently no direct medicines targeting the attenuation or reversal of liver fibrosis, and so there is an urgent need to look into this area. Traditional Chinese Medicine has a long history in using herbal medicines to treat liver diseases including fibrosis. It is time to integrate the ancient wisdom with modern science and technology to look for the best solution to the disease. In this review, the principal concept of the pathology of liver fibrosis will be described, and then some of the single compounds isolated from herbal medicines, including salvianolic acids, oxymatrine, curcumin, tetrandrine, *etc*. will be discussed from their effects to the molecular mechanism behind. Molecular targets of the compounds are analyzed by network pharmacology approach, and TGF*β*/SMAD was identified as the most common pathway. This review serves to summarize the current findings of herbal medicines combining with modern medicines in the area of fibrosis. It hopefully provides insights in further pharmaceutical research directions.

## Introduction

Liver fibrosis is a great concern in public health, as it could result in cirrhosis, portal hypertension, liver failure, and possibly hepatocellular carcinoma (HCC) that cause deaths. A common result of progressive liver fibrosis is cirrhosis, which affects 1–2% of the world population (Higashi et al., 2017); it causes over one million deaths annually ranking the 11^th^ most common cause of mortality worldwide (Asrani et al., 2019) and has an incidence of over five million in 2017 (James et al., 2018). Liver fibrosis is a chronic state of hepatic injuries, which could be the result of viral infection (HBV, HCV), alcohol consumption, drug abuse, fatty liver, steatohepatitis, as well as autoimmune disease (Seki and Schwabe, 2015). Fibrogenesis initiates with activation of effector cells by the primary injury response that leads to an elaboration and deposition of extracellular matrix. With insufficient restoration, fibrogenesis progresses and develops into organ failure.

Diverse types of cells are involved in fibrogenesis, including epithelial cells, endothelial cells, inflammatory cells, and most importantly fibrogenic effector cells. Specifically, the hepatic epithelial cells are injured by external causes and are followed by an inflammatory response. Wound healing response is stimulated and results in recruitment of inflammatory cells and activation of fibrogenic effector cells. The inflammatory cells, including Kupffer cells, mast cells, and T cells, secrete inflammation mediators and factors like chemokines and cytokines, inducing immune response with inflammation (Rockey, 2013). Those released factors could promote the activation of the fibrogenic effector cells, which is the central key in fibrogenesis, through the paracrine pathways. Fibroblasts, myofibroblasts, and certain types of cells derived from bone marrow and epithelial-to-mesenchymal transition are the crucial effectors in liver fibrosis, which are mainly responsible for producing extracellular matrix proteins (EMP) (Hinz et al., 2007). Stimulators of the cells like transforming growth factor beta (TGF*β*) are also secreted by themselves, which contribute to the autocrine activation (Meng et al., 2014). Scar tissues accumulate with EMP which are synthesized and released from the effector cells, mainly fibroblasts and myofibroblasts. The EMP include prominently collagens type I and type III, fibronectin, laminin, and some other trace amount elements (Friedman, 2008a).

**Figure 1:**
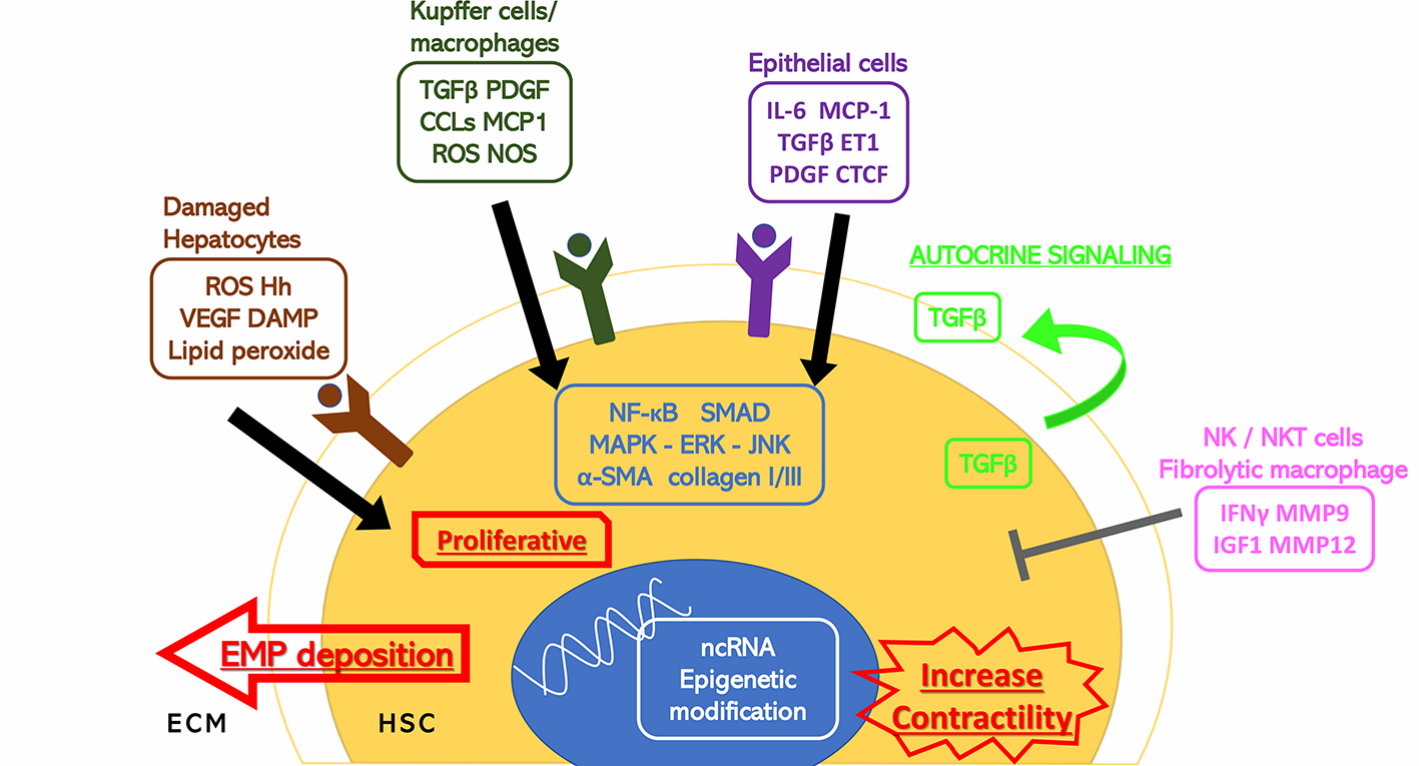
Stimuli to HSC activation. Hepatic cells surrounding the hepatic stellate cells (HSCs) including damaged hepatocytes, Kupffer cells, macrophages, epithelial cells, and natural killer/natural kill T cells have extracellular secretion to either stimulate or inhibit the activation of the HSCs through cytokines and hormones. The HSC activation response is also shown. *α*-SMA, alpha smooth muscle actin; CCL2, C-C motif chemokine 2; CTCF, connective tissue growth factor; DAMP, damage associated molecular patterns; ECM, extracellular matrix; EMP, extracellular matrix proteins; ERK, extracellular signal-regulated kinase; ET1, endothelin 1; Hh, hedgehog; HSC, hepatic stellate cell; IFN*γ*, interferon gamma; IGF1, insulin-like growth factor 1; IL-6, interleukin 6; JNK, c-Jun N-terminal kinase; MAPK, mitogen-activated protein kinase; MCP1, monocyte chemoattractant protein 1; MMP, matrix metalloproteinase; ncRNA, noncoding ribonucleic acid; NF-*κ*B, nuclear factor kappa-light-chain-enhancer of activated B cells; NOS, nitric oxide synthase; PDGF, platelet-derived growth factor; ROS, reactive oxygen species; TGF*β*, transforming growth factor beta; VEGF, vascular endothelial growth factor.

Activation of hepatic stellate cells (HSCs) from its quiescent state is an indispensable and critical step of liver fibrogenesis. HSCs are mesenchymal cells that contribute to about 15% of normal resident cells in the liver (Friedman, 2008b). They serve as the storage of vitamin A (retinoid) in normal liver, but this characteristic is deprived once the HSCs start to transdifferentiate under the stimulation of cellular mediators, cytokines, and chemokines from injured or inflammatory cells. At the initiating stage of fibrogenesis, HSCs are activated to become proliferative and contractile myofibroblasts. The transdifferentiated HSCs accelerate the secretion and attenuate the degradation of the extracellular matrix elements, which eventually lead to fibrogenesis (**Figure 1**). Studies showed that selective inhibition of HSCs exhibited prominent potential in preventing and reversing fibrogenic process in rodents (Marti-Rodrigo et al., 2019; Yan et al., 2019).

Current therapeutic strategy on liver fibrosis is to remove and eliminate the etiology. Until now, there are no “golden standard” therapies for liver fibrosis. However, accumulating preclinical evidence has suggested that the scarring process of the liver is not unidirectional and permanent, but instead plastic and reversible (Campana and Iredale, 2017; Higashi et al., 2017; Zoubek et al., 2017; Schuppan et al., 2018). Owing to the primary role in mediating fibrogenesis in the liver, HSCs have become arousing interesting drug targets to the prevention and treatment of hepatic fibrosis (Lotersztajn et al., 2005). Inhibition of HSCs could be achieved by reversing transdifferentiation of HSCs into myofibroblast, reducing the fibrogenic activity of the HSCs, and inducing death or apoptosis of HSCs. Natural products derived from medicinal plants and animals, such as silymarin, catechins, schisantherin, and ursodeoxycholic acid, have shown proofs of beneficial effects and were approved as healthy supplements for patients with chronic liver diseases (Paumgartner and Beuers, 2002; Levy et al., 2004; Hong et al., 2017; Bagherniya et al., 2018; Daniyal et al., 2019). Moreover, a great number of herbal medicine and bioactive compounds are under investigation for their antifibrotic activity. In this review, we summarized the current research progress on compounds isolated from herbal medicine in treating liver fibrosis by targeting HSCs. We searched through the PubMed database with the keywords “liver fibrosis”, “hepatic stellate cells”, and “herbal medicine”. We aim to highlight the role of herbal drugs in modern medicine and provide insights and perspectives on the research and development of first-line fibrosis therapies targeting hepatic stellate cells.

## Hepatic Stellate Cells as Therapeutic Targets of Liver Fibrosis

### Activation of Hepatic Stellate Cells From Their Quiescent State

The first step of HSCs involvement in fibrosis is the initiation stage, where there are primary modulations in genetic expressions and phenotypic changes sensitized by paracrine cytokine and chemokine stimulation. HSCs locate in the perisinusoidal space where neighboring cells including Kupffer cells, hepatocytes, and endothelial cells could cause reshaping in the microenvironment (Wake, 1971). Cytokines, mainly platelet-derived growth factor (PDGF), TGF*β*, interleukin 1 beta (IL-1*β*), tumor necrosis factor (TNF), monocyte chemoattractant protein 1 (MCP1), C-C motif chemokine (CCL)-3, CCL5, are secreted by Kupffer cell (Marra et al., 1993; Pinzani, 2002; Pradere et al., 2013). Damage-associated molecular patterns (DAMP), reactive oxygen species (ROS), and such inflammatory mediators are released from injured hepatocytes which trigger innate immune response. Hepatic nuclear factor *κ*B (NF-*κ*B)-inducing kinase is activated, while lipid peroxides and TNF-related apoptosis-inducing ligand (TRAIL), hedgehog ligands, and so forth are released from leukocytes (Canbay et al., 2002; Shen et al., 2014; Lan et al., 2015). These can cause activation of the HSCs, resulting in matrix synthesis, proliferation, and loss of retinoids. Instead of causing damage to normal hepatocytes, those activators induce the transdifferentiation of the quiescent HSCs into activated form. Carbon tetrachloride (CCl_4_)-induced liver injury model in rodent showed the HSC activation and liver fibrosis mechanism (Miao et al., 2019). The free radical product CCl_3_ produced by the cytochrome CYP2E1 in liver cells leads to elevated activation of HSCs (Bedossa et al., 1994). Depletion of macrophages by transgenic means and administrated liposomal clodronate caused suppressed HSC activation and fibrogenesis in CCI_4_ chronic hepatic injury mice model (Duffield et al., 2005; Sunami et al., 2012).

Subsequently, the HSCs would enter into the second stage, which was first named “perpetuation” by Friedman twenty years ago (Friedman, 2000). In this stage, the phenotypes of the activated HSCs are amplified and results in elevated proliferation, scar formation, contractility, reduced matrix degradation, and fibrogenesis (Iwaisako et al., 2014). The apparent net change of these behaviors is the accumulation of extracellular matrix.

### Major Factors Involved in Hepatic Stellate Cell Activation

While many factors are able to activate the HSCs from their quiescent state in *in vitro* studies, there are two major cytokines proven to be the dominant inducers that lead to HSC activation *in vivo*. The sustained interaction between HSCs and paracrine and autocrine TGF*β* and PDGF in the hepatic microenvironment results in consecutive activation of the cells throughout the initiation and progression of liver fibrosis.

#### Transforming Growth Factor Beta

TGF*β* has long been identified as one of the most potent cytokines to induce fibrogenesis (Hellerbrand et al., 1999). HSCs are activated by signals from the TGF*β*, but at the same time they secrete TGF*β*, which completes an autocrine positive feedback mechanism. The direct downstream effector of the signaling pathway is the SMAD proteins, predominantly SMAD2 and SMAD3 (Shi et al., 2011). The binding of TGF*β* to its type 1 receptor (TGF*β*R1) brings phosphorylation to the receptor and thus the SMAD2/3 proteins. The p-SMAD proteins then bind to SMAD4, which forms a complex translocating to the nucleus. It could affect the epigenetic modifications, noncoding RNA (ncRNA) expressions, as well as the induction of myofibroblast and matrix deposition (Meng et al., 2016). TGF*β* may also act on the mitogen-activated protein kinase (MAPK) pathway, with extracellular signal-regulated kinase (ERK), p38, c-jun N-terminal kinase (JNK) as downstream cascades (Engel et al., 1999; Hanafusa et al., 1999). Alpha-smooth muscle actin (*α*-SMA), EMP like fibronectin, proteoglycans (George et al., 2000), and especially collagen types I and III are upregulated through induced transcription (Breitkopf et al., 2006). *α*-SMA induction is one of the critical markers demonstrating HSC activation (Tomasek et al., 2002) due to its absence in the surrounding resident hepatocytes except the smooth muscle cells inside blood vessels (Friedman, 2008a). Extracellular collagen type I and III levels are elevated in fibrotic liver, whereas type I is the most characteristic one leading to cirrhosis (Rojkind et al., 1979). It was elucidated that the augmentation of collagen I by TGF*β* stimulation is dependent of the mediator hydrogen peroxide and the CCAAT/enhancer binding protein-*β* (C/EBP*β*) (Garcia-Trevijano et al., 1999).

#### Platelet-Derived Growth Factor

Animal studies have shown the critical role of the mitogen PDGF as well as its receptor PDGF receptor-*β* (PDGFR*β*) in HSC proliferation and migration (Wong et al., 1994; Kostallari et al., 2018). The extent of inflammation so as fibrosis is correlated with the expression of PDGF in patients with chronic liver diseases (Zuo et al., 2019). Interestingly, the mRNA expression of PDGFR*β* was confirmed in both quiescent and activated HSCs, but protein production was mainly limited to the activated cells (Henderson et al., 2013). The PDGF-induced proliferation could be attenuated by an adipocytokine adiponectin (Kamada et al., 2003), whereas leptin had the opposite effect (Saxena and Anania, 2015).

#### Vascular Endothelial Growth Factor

The vascular endothelial growth factor (VEGF) induces cell proliferation especially HSCs, which includes angiogenesis in the damaged liver tissue. It has a complicated role which takes part in both fibrogenesis and hepatic tissue repair and reversal of fibrosis (Kantari-Mimoun et al., 2015). VEGF may be a pathological factor in the induction of HSC activation, in hypoxic environment (Ankoma-Sey et al., 2000), but it also regulates liver sinusoidal permeability, monocyte migration, and scar-associated macrophage function, which are fibrotic resolution and tissue repair processes (Yang et al., 2014).

#### Connective Tissue Growth Factor

The connective tissue growth factor (CTGF) is highly expressed in fibrotic liver when compared to normal liver. It is a potent fibrogenic cytokine similar to PDGF. Its contribution to ECM accumulation brings about a series of hepatic fibrogenic actions (Huang and Brigstock, 2012). CTGF activates and at the same time is produced mainly by HSCs. It is particularly important because it is one of the primary drivers to fibrillar collagen productions. CTGF expression is reported to be associated with the microRNA miR-214 in an inverse proportion (Chen et al., 2014a; Chen et al., 2015).

#### Hedgehog Pathway

The hedgehog (Hh) pathway is an essential system in the regulation of progenitor cells’ fate in the fibrosis of liver. Smoothened homolog (SMO), which is released and activated with the upregulation of Hh ligands, drives the epithelial regeneration by promoting mesenchymal-to-epithelial transitions of the myofibroblasts derived from HSCs (Omenetti et al., 2011). Mice experiments have demonstrated that the deletion of SMO could attenuate fibrogenesis in liver injury models. Other studies also proved that the blockade of Hh signaling could inhibit the liver fibrosis and reduce liver progenitor cells (Greenbaum and Wells, 2011). The Hh pathway could possess the possible targets of fibrotic treatment (Shen et al., 2017).

#### Toll-Like Receptor

Dietary or free cholesterol in the liver could worsen fibrosis by activating HSCs. The elevated intracellular cholesterol level in HSCs leads to Toll-like Receptor (TLR) 4 signaling (Teratani et al., 2012). The accompanying result is the sensitization of HSC to TGF*β*-activation by the reduction of TGF*β* pseudoreceptor bone morphogenetic protein and activin membrane-bound inhibitor (Bambi). The deficiency of a cholesterol acyltransferase accelerates the fibrosis develop through the insufficient removal of free cholesterol in HSCs (Tomita et al., 2014). Therefore, cholesterol-lowering drugs could help alleviate the fibrosis by slowing down the accumulation of free cholesterol (Van Rooyen et al., 2013).

### Molecular Strategies of Hepatic Stellate Cell Suppression

Despite the advancement of effective antiviral agents that could target the underlying causes of the fibrotic result by hepatitis B and C (Schuppan et al., 2018), there are some other etiologies of these liver diseases including alcoholic and nonalcoholic steatohepatitis, autoimmune diseases, *etc*. that remain poorly solved. A viable therapeutic approach is arising with HSCs as the target. Since HSCs is the major mediator in the process of fibrogenesis, reducing the activity of HSCs could slow down or possibly revert the fibrosis condition. On the purpose of HSCs regression and clearance, currently there are three therapeutic approaches, namely apoptosis, senescence, and reversion.

#### Apoptosis

The transdifferentiated HSCs express antiapoptotic activity under proinflammatory stimuli TNF and IL-1*β* through the NF-*κ*B signaling pathway (Pradere et al., 2013), and the production of antiapoptotic proteins like Bcl-2 is the result (Lee et al., 2015). Tissue inhibitors of metalloproteinase 1 (TIMP-1) and TGF*β* also promote antiapoptotic signals and survival of HSCs (Murphy et al., 2002). As such, the treatment on the HSCs should induce susceptibility to cell death in order to reduce the number of transdifferentiated HSCs. The activated HSCs have receptors such as apoptosis antigen 1 (FAS, CD95), TNF receptor 1 (TNFR1), TRAIL receptors, and p75 neurotrophin receptor (p75NTR), which stimulate apoptosis when engaged (Pellicoro et al., 2014). NF-*κ*B inhibitor BAY 11-7082 and proteasome inhibitors bortezomib and MG132 can inhibit the NF-κB gene and so its pathway on the HSCs, which can in turn reduce liver fibrosis (Anan et al., 2006). Natural killer cells (NK) also play an essential role in the induction of HSC apoptosis (Heymann and Tacke, 2016). Interferon gamma (IFN*γ*) is extensively produced by NK, which can block the HSCs activation. This cytokine can also enhance the cytotoxicity of NK on HSCs by increasing NKG2D and TRAIL related apoptosis induction (Radaeva et al., 2006). Target-constructed-IFN*γ* could also bind to the PDGFR*β* on the HSCs to cease activation and induce fibrolysis (Bansal et al., 2011). Sorafenib is a first-line tyrosine kinase inhibitor that is used to treat renal cell and hepatocellular carcinoma (HCC) (Lyons et al., 2001). It also shows inhibitory effect and induces autophagic cell death on HSCs through the Akt/mTOR/p70S6K and JNK signaling pathways (Hao et al., 2016).

#### Senescence

When cell proliferation exceeds a finite number of times, cellular senescence occurs, and the cell-cycle would arrest to prevent genetic damages. Senescence is mainly mediated by the p53-dependent pathway, and the attenuation of this program in HSCs enhances liver fibrosis and exacerbates the chances of developing into HCCs (Lujambio et al., 2013). IL-6 and IFN*γ* is normally secreted in senescent p53 pathway from the HSCs, while IL-3, IL-4, and IL-5 from proliferating HSCs stimulate M2-polarization of macrophages that can promote malignant cell growth. The CCN family matricellular proteins, cysteine-rich protein 6_1_ (CCN_1_/CYR6_1_) contribute to HSC senescence and apoptosis by attenuating the TGF*β* signaling (Borkham-Kamphorst et al., 2014). OSU-03012, which is a celecoxib derivative, can suppress the proliferation of HSCs and result in senescence (Zhang et al., 2015). During activation of HSCs, the retinol storage is depleted and the level of peroxisome proliferator-activated receptor gamma (PPAR*γ*) is decreased (Lee and Jeong, 2012). So, retinoid receptor and PPAR*γ* agonists are suggested to have a role in antifibrotic treatment (Panebianco et al., 2017).

#### Reversal of the Activation

Evidences have shown that activated HSCs could be reverted to a quiescent-like but distinct form. When the stimuli for transdifferentiation were removed from the rodent models, half of the myofibroblasts escaped from apoptosis, with fibrogenic genes downregulated and reverted phenotypes. The HSCs remained in an inactivated state until resensitized by fibrogenic stimuli again. The reverted HSCs were not completely identical to the preactivated quiescent ones, with fibrogenic genes as well as quiescence-associated genes expressed in a lesser extent. (Kisseleva et al., 2012; Troeger et al., 2012). A recent study has revealed the possibility of reprogramming the profibrogenic myofibroblasts into hepatocyte-like cells, named induced hepatocytes (iHeps). Using chronic liver disease mouse models, liver myofibroblasts were treated with a set of transcription factors including FOXA3, GATA4, HNF1A, and HNF4A and resulted in ameliorated liver fibrosis. Most iHeps were found near the portal vein and central vein regions, and they assisted in restoring the deprived liver function (Song et al., 2016).

## HSC-Targeting Natural Compounds from Herbal Medicine for Liver Fibrosis Treatment

Although there are currently countable numbers of approaches on the treatment targeting HSCs undergoing clinical trials, the use of herbal drugs or the isolated active single compounds are also worth investigating due to its cheap cost and low risk of side effects. There has been a long history in using herbal medicines and natural compounds in treating liver diseases. In the following, plenty of small molecular compounds derived from herbal medicines are discussed in detail and summarized in **Table 1**.

**Table 1:** Molecular mechanism of compounds isolated from herbal medicine on hepatic stellate cells against liver fibrosis.

Compounds	Effects	References
Salvianolic acid A	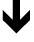 lipid peroxidation, ALT, AST, deposition of collagens I and III	(Liu et al., 2000)
	Induce apoptosis and inhibit activation of HSC, through depression of Bcl-2, cyclin D1, E, phosphorylation of AKT and PDGF, and elevated p21 and p27	(Lin et al., 2006)
	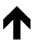 liver function 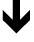 Hyp and MDA content	(Liu et al., 2001)
	*α*-SMA and TGF*β*	(Qiang et al., 2014)
	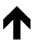 HSF1, regulate SIRT1 pathway	(Zhu et al., 2018)
Salvianolic acid B	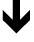 cyclooxygenase activity	(Liu et al., 1994)
	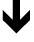 ALT/AST and total bilirubin, reverse fibrotic score in clinical trial	(Liu et al., 2002a)
	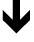 HSC proliferation, cell cycle arrest at G1-S phase	(Cui et al., 2002; Yang et al., 2003)
	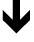 PDGF stimulation through MAPK signaling	(Liu et al., 2002b)
	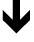 SMAD2/3 protein activity	(Wu et al., 2019a)
	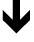 H_2_O_2_ induced mitochondrial dysfunction	(Liu et al., 2017)
	Regulate NF-*κ*B/I-*κ*B-*α* pathway	(Wang et al., 2012)
Oxymatrine	Antihepatitis B and C virus	(Wu and Wang, 2004; Wang et al., 2011)
	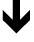 IL-6, TNFα, SMAD3, CREBBP, TLR4 *via* TGF*β* pathway 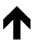 IL-10, Bambi, SMAD7	(Zhao et al., 2016)
	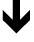 SMAD3 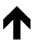 SMAD7	(Wu et al., 2005; Wu et al., 2008)
	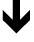 HSC-T6 cell 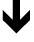 miR-195 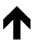 SMAD7	(Song et al., 2019)
	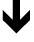 procollagen I YB-1 nuclear translocate Regulate ERK1/2 pathway	(Du et al., 2015)
	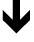 TIMP-1	(Shi and Li, 2005)
	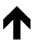 effect with RGD-liposome	(Chai et al., 2012)
Curcumin	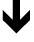 ALT, TGF*β*	(Reyes-Gordillo et al., 2008)
	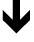 PDGFR*β*, ERK, serum PDGF, CTGF 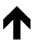 MMP-9, ECM degradation	(Zhang et al., 2012)
	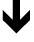 *α*-SMA, TGF*β*, SMAD2, SMAD3, CTGF 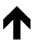 SMAD7	(Yao et al., 2012)
	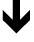 TNF*α*, IL-6, MCP-1, HMGB-1, TLR4, TLR2, activated HSC	(Tu et al., 2012)
	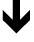 ALT, AST, TGFα 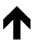 MMP-13, reduced GSH	(Morsy et al., 2012)
	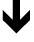 Collagen deposition, NF-*κ*B, TNF*α*, IL-1*β*, IL-6 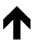 IL-10	(Wu et al., 2010)
	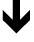 TNFα, NF-*κ*B, IL-6	(Bassiouny et al., 2011)
	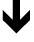 the leptin activation on HSC 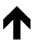 PPAR*γ*	(Tang et al., 2009)
	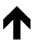 AMPK activity 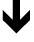 HSC activation	(Tang and Chen, 2010)
	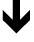 GLUT-2 Disrupt p38 MAPK pathway 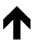 PPAR*γ*	(Lin and Chen, 2011)
Tetrandrine	Block calcium ion channel	(Bataller et al., 1998)
	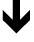 collagen deposition, *α*-SMA, DNA synthesis	(Park et al., 2000)
	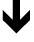 Hyp, *α*_1_-collagen and TIMP-1	(Lee et al., 2001)
	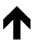 HSC apoptosis	(Yin et al., 2007)
	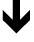 TGF*β* 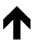 SMAD7	(Chen et al., 2005)
	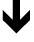 TNF-α, NF-*κ*B, *α*-SMA, collagen deposition 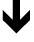 Phosphorylation of I*κ*B*α*, ICAM-1 expression	(Hsu et al., 2007)
	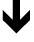 *α*-SMA, TRADD, TAK1, p-JNK 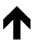 I*κ*B*α*, JNK, NF-*κ*B, p-ERK, caspase-3, PARP	(Li et al., 2016)
	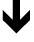 autophagic, fibrogenic signals 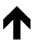 lipid accumulation	(Miyamae et al., 2016)
Quercetin	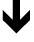 TGF*β*, ECM, collagen I, *α*-SMA, p-SMAD2/3 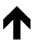 PI3K, p-Akt	(Wu et al., 2017)
Artesunate	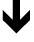 Hyp, MMP-2, MMP-9, *α*-SMA, collagen I 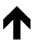 MMP-13	(Xu et al., 2014)
	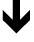 p-FAK, Akt, GSK-3*β*	(Lv et al., 2018)
	Induce ferroptosis in HSC	(Kong et al., 2019)
Glycyrrhetinic acid	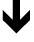 Collagen I, nuclear SMAD3, COL1A2	(Moro et al., 2008)
	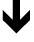 cleaved caspase-3, Bax, CTGF, *α*-SMA, collagen I and III, MMP-2, MMP-9	(Liang et al., 2015)
	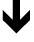 Inflammation, Hyp, *α*-SMA, collagen I, TGF*β*1, SMAD2/3, SMAD3 mRNA, p-SMAD2/3	(Zhou et al., 2016)
Resveratrol	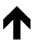 SOD, MDA, ATPase	(Ahmad and Ahmad, 2014)
	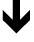 protein carbonyls, *α*-SMA, collagen deposition	Zhang et al., 2016
	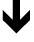 cell viability, *α*-SMA, collagen I, TLR4, MyD88, PI3K, Akt, translocation of NF-κb	(Zhang et al., 2016)
Deoxyschizandrin	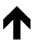 CYP3A4, CYP3A5	(Yang et al., 2015)
	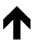 LC3-II 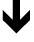 p62, beclin 1	(Lu et al., 2014)
Ligustrazine	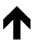 PPAR*γ* 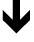 HIF-1*α*, VEGF, bFGF, ICAM-1, VCAM-1, p-MLC2, migration and adhesion of HSC	(Zhang et al., 2018)
	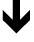 SMO, Gli 1, bcl-2, cyclin-D1, HSP90, HIF-1*α*, VEGF, angiopoietin 1	(Zhang et al., 2017)
Astragaloside	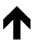 MMP-2, GSH, SOD, p-SMAD2, p-SMAD3 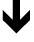 TGF*β*1, SMAD7, collagen I, collagen III, TIMP-2, *α*-SMA, MDA	(Yuan et al., 2018)
	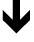 *α*-SMA, collagen I	(Guo et al., 2018)
	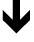 AST, ALT, TNF-α, IL-6, IL-1, p-PI3K/PI3K, p-Akt/Akt, p-mTOR/mTOR	(Wei et al., 2019)
Paeonol	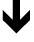 HSC migration, *α*-SMA, collagen, p-p38, p-ERK, p-JNK, p-PDGFR*β*	(Kuo et al., 2012)
	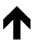 GSH-PX, SOD, CAT 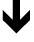 TGF*β*, SMAD3, ALT, AST, Hyp, IL-6, TNF-α, MDA, collagen 1a, *α*-SMA, vimentin, desmin	(Wu et al., 2019b)
	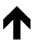 Bax, cleaved caspase-9 & caspase-3, cleaved PARP 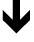 p-NF-*κ*B, I*κ*Bα, ALT, AST, Hyp, *α*-SMA, collagen 1a, CTGF, bcl-2,	(Kong et al., 2013)

### Salvianolic Acids

Salvianolic acids (SAs) are water-soluble extracted compounds isolated from the dried root of *Salvia miltiorrhiza* Bunge *(Radix Salviae miltiorrhizae)*, a herbal TCM medicine, *Danshen* that have been used thousands of years for the treatment of cardiovascular diseases (Li et al., 2018). With modern understanding in chemistry and pharmacology, SAs are also demonstrated to have observable antioxidative and anticancer effects. Moreover, SAs can modulate fibrogenesis through signal transduction.

**Figure 2:**
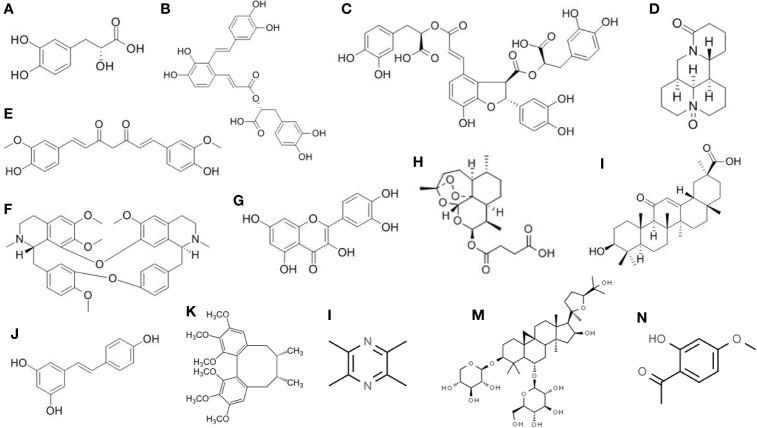
Chemical structure of the isolated active compounds from herbal medicine with therapeutic effects on liver fibrosis. **(A)** Denshensu. **(B)** Salvianolic acid A. **(C)** Salvianolic acid B. **(D)** Oxymatrine. **(E)** Curcumin. **(F)** Tetrandrine. **(G)** Quercetin. **(H)** Artesunate. **(I)** Glycyrrhetinic acid. **(J)** Resveratrol. **(K)** Deoxyschizandrin. **(L)** Ligustrazine. **(M)** Astragaloside. **(N)** Paeonol.

Traditionally, *S. miltiorrhiza* was used to enhance blood circulation, attenuate congestion, and modulate menstrual cycle. It is also commonly used in modern Chinese Medicine to treat ischemic stroke, atherosclerosis, viral myocarditis, chronic hepatitis, cancers, as well as liver fibrosis (Zhou et al., 2005; Liu et al., 2010; Zhu et al., 2018). Among the water-soluble compounds in *S. miltiorrhiza*, SAs have the highest solubility (Liu et al., 2007). Until now there are more than 10 different types of SAs identified, which are coded as salvianolic acid A, B, C, D, *etc*. (Ma et al., 2019). Salvianolic acid A (SAA) and salvianolic acid B (SAB) are the most abundant ones in the extract. All the salvianolic acids are possessing a common subunit Danshensu [(*R*)-3-(3, 4-Dihydroxyphenyl)-2-hydroxypropanoic acid] (Chen et al., 2014b) (**Figure 2**). SAA is composed of one Danshensu unit, while SAB is composed of three. SA showed higher anti-inflammatory and antioxidative activity than other compounds in *S. miltiorrhiza* (Du et al., 2016).

There are recent studies showing the antifibrosis effect of SA, particularly on liver fibrosis. These effects are inevitably related to the inhibition of HSC activation or the induction of apoptosis of HSCs. SAAs are believed to suppress lipid peroxidation, reduce alanine aminotransferase (ALT) and aspartate aminotransferase (AST) activity, as well as deprive the deposition of collagens I and III (Liu et al., 2000). HSCs induced apoptosis and inhibited activation by SAA, through the depression level of Bcl-2, cyclin D1, and E proteins, phosphorylation of AKT and PDGF, with elevated expression of p21 and p27 (Lin et al., 2006). Liver function was repaired; hydroxyproline (Hyp) and malondialdehyde (MDA) contents were attenuated by SAA in a rat model (Liu et al., 2001). In streptozotocin-induced diabetic rat model, SAA has shown to slow down the progression of liver fibrosis by reducing the expression of *α*-SMA and TGF*β* (Qiang et al., 2014). SAA was also suggested to have a protective effect against bile duct ligation induced liver fibrosis through sirtuin 1 (SIRT1)/heat shock factor 1 (HSF1) signaling pathway (Zhu et al., 2018). Endoplasmic reticulum stress was abrogated by increased HSF1 expression.

SAB has all the aforementioned effects on countering fibrosis like the SAA (Luk et al., 2007; Li et al., 2012). Moreover, it was reported that SAB could also inhibit the cyclooxygenase activity in rat liver (Liu et al., 1994). SAB was proved to have a reversal effect on fibrosis in a double-blinded randomized control clinical study (Liu et al., 2002a). It was reported that SAB has as much, if not more, therapeutic effects and anti-inflammatory effects compared to IFN*γ*. Up to 10 µM of SAB could result in inhibition of cell proliferation and cell cycle arrest at G1 to S phase (Cui et al., 2002; Yang et al., 2003). SAB is, at the same time, limiting the PDGF stimulation through MAPK activity (Liu et al., 2002b). This mediation is suggested to be of the reduction of TGF*β*-induced HSCs undergoing SMAD signaling pathway. SAB suppressed SMAD2/3 protein phosphorylation at the linker region of SMAD2/3 and the C-terminal in SMAD2, while it increased at the C-terminal in SMAD3 (Wu et al., 2019a).

### Oxymatrine

Oxymatrine (OM) is one of the many effective quinolizidine alkaloids extracted from the herbal medicine root of *Sophora flavescens* Aiton *(Kushen)*. It is an oxygenated form of another alkaloid matrine. *Kushen* has been traditionally used with other herbal medicines together to treat fever, hematochezia, dysentery, jaundice, oliguria, *etc*. (He et al., 2015). Its extracted active compound, OM, has the effect of antiarrhythmia, myocardial ischemia prevention, prophylactic, anti-inflammation and reducing oversensitivity (Deng et al., 2019). In recent years, its ability to attenuate fibrogenesis and carcinogenesis is being widely studied (Halim et al., 2019; Lan et al., 2019).

OM was proved to be antihepatitis B and C virus, which is effective to reduce viral-caused hepatitis and fibrosis (Wu and Wang, 2004; Wang et al., 2011). It is also effective against fibrosis from other causes. OM attenuated liver fibrosis by limiting the CCI_4_-induced proinflammatory cytokines IL-6 and TNFα while promoting IL-10 and Bambi such anti-inflammatory factors (Zhao et al., 2016). It was suggested that OM modulates the HSC activation by suppressing TLR4 *via* the TGF*β* signaling pathway. Collagen deposition in the liver is significantly reduced by OM-treated rats, which is accompanied by an elevation of SMAD7 and inhibition of SMAD3 as well as cAMP-response element-binding protein binding protein (CREBBP). This is consistent with the modulation of the fibrogenesis *via* TGF*β* pathway with SMAD as the downstream effecter (Wu et al., 2008). OM was observed to have a similar effect on the pig serum-induced liver rat fibrosis (Wu et al., 2005).

The molecular mechanisms of OM on HSCs were studied *in vitro*. It was found that OM has an inhibitory effect on HSC-T6 cell line *in vitro* with concentration higher than 200 μg/ml after 24 h and 10 μg/ml after 72 h. miR-195, which is essential in HSC activation, was significantly down-regulated. SMAD7 level was augmented in the meantime (Song et al., 2019). Another study working on the LX-2 human HSC line has shown the possible mechanism of reducing procollagen I expression (Du et al., 2015). A transcription factor Y-box binding protein 1 (YB-1) was observed to have nuclear translocation under OM treatment at a concentration of 960 mg/L. The phosphorylation level of ERK1/2 was found to be positively associated with YB-1 expression. Thus, it is suggested that OM also regulates HSC activity *via* the ERK1/2 signaling pathway. The expression level of TIMP-1 was significantly lowered by OM in a CCI_4_-induced fibrosis rat model, with no differences in *α*-SMA expression between the control or treatment group (Shi and Li, 2005). The therapeutic effect of OM on HSCs was reported to be enhanced using the Arg-Gly-Asp (RGD)-mediated targeting delivery liposome (Chai et al., 2012). This combined formulation could increase the inhibitory effect of OM on hepatic fibrosis by reducing HSC viability, inducing apoptosis, and limiting fibrogenesis gene expressions.

### Curcumin

Curcumin is the principal phenol found in the rhizome of the herb *Curcuma longa* L., or turmeric as the common name, which has been a Chinese medicinal herb *Jianghuang*. It is a kind of curcuminoid, which is responsible for most of the biological activity of *C. longa*. In traditional Chinese medicine, it has been used in the treatment of chest and gut pain, dysmenorrhea, abdominal mass, wound healing, as well as rheumatic numb and pain. In modern pharmacology, curcumin was reported to have anti-inflammatory, antioxidant, antimicrobial, chemopreventive, chemotherapeutic, and anticancer activity (Anand et al., 2007; Hatcher et al., 2008).

The major effect of curcumin on liver fibrosis has been extensively studied, and it has been documented that the mechanism is a major target on HSC activation. There are many studies proving the effect of curcumin on HSCs, and this has been a potential therapeutic approach to be researched. There are reported evidences that TGF*β* (Reyes-Gordillo et al., 2008), PDGFR*β*, serum PDGF, CTGF (Zhang et al., 2012), SMAD2-3 (Yao et al., 2012), TNFα (Tu et al., 2012), matrix metalloproteinases (MMPs) (Morsy et al., 2012), TLRs (Tu et al., 2012), and some inflammatory cytokines (Wu et al., 2010; Bassiouny et al., 2011) are targeted and attenuated.

As mentioned previously leptin is a mediator in the development of liver fibrosis, especially in patients with obesity and type II diabetes mellitus (Stefanovic et al., 2008; Watanabe et al., 2008). During HSC activation, the cellular lipid storage is depleted, and lipid accumulation related gene expressions are downregulated. Leptin as a hormone in regulating lipid metabolism and energy balance (Friedman, 2004) was shown to stimulate HSC activation during fibrogenesis (Aleffi et al., 2005; Cayon et al., 2006). However, it is suggested that curcumin could revert this stimulation action by leptin on HSCs. Curcumin was demonstrated to have an inhibitory effect on leptin activation by reducing the phosphorylation level of the leptin receptor while stimulating PPARγ activity and leading to the interruption of leptin signaling (Tang et al., 2009). In another study performed by the same group later in 2010, curcumin was found also to attenuate the effect of leptin by promoting the activity of AMP-activated protein kinase (AMPK). The increased AMPK level results in induction of expression of lipid accumulation genes, which in turn slows down the HSC activation (Tang and Chen, 2010).

On the other hand, there are arising evidences showing that high blood glucose or the state of hyperglycemia could lead to the activation of HSCs (Aggarwal, 2010). Curcumin could restore this situation (Lin and Chen, 2011). The phytochemical could suppress the translocation of glucose transporter (GLUT)-2 to the cell membrane by disrupting the p38 MAPK pathway. The GLUT2 gene expression was downregulated by stimulating PPARγ activity. Less glucose could be imported by HSCs, and the diabetes-associated hepatic fibrogenesis was under control.

### Tetrandrine

Tetrandrine (Tet) is an alkaloid extracted from the herbal medicine, *Stephania tetrandra* S. Moore, or called *Fangji* in Chinese Medicine, which acts on the calcium ion channel (Li et al., 2001). *Fangji* has been prescribed to treat rheumatic diseases, hypertension, numbness, edema, urination problems, and sores traditionally. In modern Chinese Medicine, Tet is also used for the purpose of analgesic, anti-inflammation, treating tuberculosis, lowering blood pressure, antimyocardial ischemia, antiarrhythmia, antifibrosis, and anticancer *etc*. (Yin et al., 2007; Bhagya and Chandrashekar, 2018).

The antifibrosis effect of Tet is due to its function of abrogating HSC activation as well as inducing HSC apoptosis. It has a blocking action of the calcium ion channels on the HSCs, which could suppress their contractility and thus the activation (Liu et al., 1995; Bataller et al., 1998). Tet was shown to reduce collagen deposition in ECM in liver fibrosis induced by bile duct ligation and scission in rat (Park et al., 2000). Reduced *α*-SMA was observed in the HSCs, while DNA synthesis was also attenuated. Another study suggested that Tet reduced liver Hyp content through reducing the *α*_1_-collagen and TIMP-1 mRNA level (Lee et al., 2001). HSC apoptosis could also be the result of Tet treatment as described by (Yin et al., 2007). The effect has almost no differences when compared with the group treated with IFN*γ*. Tet has a similar suppressive effect on TGF*β* and an inductive effect on SMAD7, which results in reduced activation of HSCs (Chen et al., 2005).

A more detailed mechanism was illustrated in 2007, where Tet inhibition on TNF-α-induced NF-κ*B* transcription was found to be concentration dependent (Hsu et al., 2007). In addition, Tet attenuates TGF*β* induced *α*-SMA production and collagen deposition in cultured HSC-T6 cells. Phosphorylation of I*κ*B*α* and ICAM-1 expression is reduced, resulting in a total decrease of activated *α*-SMA positive HSCs. Tet was also reported to have the counter effect to TNF-α on HSCs activation. With dose-dependent effect, Tet could attenuate the *α*-SMA and TNF-receptor-1-associated death domain (TRADD) expression. It also has the inhibitory effect on the TGF*β*-activated kinase-1 (TAK1) and JNK phosphorylation. Furthermore, the phosphorylation of NF-*κ*B and degradation of I*κ*B*α* was suppressed by Tet treatment (Li et al., 2016). Apoptosis of HSCs was confirmed by the increased level of caspase-3 and poly (ADP-ribose) polymerase (PARP) at higher concentration (>12.5 μM) of Tet. A more recent study suggested another role of Tet in the deactivation of HSCs (Miyamae et al., 2016). As discussed above, there exists lipid degradation during HSC activation. Tet was found to inhibit the degradation of the lipid droplets as autophagic cargo and induce lipid accumulation which makes the HSC-T6 cell line remain quiescent. This result suggested that Tet could also target the late autophagy regulators.

### Other Compounds From Herbs or Plants

Quercetin is a common flavonoid found in many fruits and vegetables such as onions, kales, green tea, apples, berries, *etc*., which has been used as a supplement for its antioxidant and anti-inflammatory effects. There are researches suggesting its potential in preventing hepatic fibrosis. It was reported that quercetin could inhibit HSC activation and possibly reduce autophagy by acting on the TGF*β*1/SMAD signaling pathway, as well as activating the phosphoinositide 3-kinase (PI3K)/Akt pathway. ECM, collagen I, and *α*-SMA production is inhibited, MMPs are increased by quercetin on the CCl_4_ fibrosis model, and TIMP-1 was upregulated (Wu et al., 2017). Quercetin derivatives have also similar antifibrotic effects as well. By adding methyl group to a different position on the quercetin, there are different singular effects against various features of fibrosis respectively. This provides potentials in studying the enhancement of quercetin’s therapeutic effect (Ganbold et al., 2019).

Artesunate is a semisynthetic derivative of the artemisinin group of drugs that is most commonly used to treat malaria. Artemisinin was isolated from the Chinese medicine *Artemisia annua* L. by the 2015 Nobel Prize laureate Tu Youyou. Artesunate was found to have the attenuation effect on liver fibrosis. Hyp content was significantly decreased; MMP-2, MMP-9, *α*-SMA, and collagen I were inhibited in a bovine serum albumin induced fibrosis rat model. MMP-13 level was promoted by artesunate, and it can be concluded that it is inhibiting the activation of HSC (Xu et al., 2014). Artemisinin was also proved to reduce the phosphorylation level of focal adhesion kinase (FAK), Akt, as well as glycogen synthase kinase 3 beta (GSK-3*β*). HSC proliferation and activation are inhibited by artesunate, and apoptosis is promoted through the FAK/Akt/*β*-catenin pathway (Lv et al., 2018). One study suggested that artesunate could also bring antifibrosis effect by inducing ferroptosis in activated HSCs (Kong et al., 2019).

Glycyrrhetinic acid is a main active compound from the herbal medicine *Glycyrrhiza uralensis* Fisch. ex DC., or *Gancao* in Chinese. It was shown to significantly inhibit liver fibrosis induced by CCl_4_. It has a similar effect in cultured HSCs, that collagen I, nuclear accumulation of SMAD3, and alpha2(I) collagen gene (COL1A2) are abolished (Moro et al., 2008). It was confirmed again that hepatocyte apoptosis, *i.e.* cleaved caspase-3, Bax, CTGF, and HSC activation, *i.e. α*-SMA, collagens I and III, MMP-2, MMP-9, were all decreased by treatment of glycyrrhetinic acid (Liang et al., 2015). In addition, it was suggesting that a combination of glycyrrhetinic acid and astragalus saponins, components isolated from *Astragalus mongholicus* Bunge *(Huangqi)*, could effectively reduce liver inflammation, ECM deposition, and HSC activation in liver fibrosis rats. The combined therapy significantly reduced SMAD3 mRNA, TGF*β*1, SMAD3, and p-SMAD2/3 protein levels, when compared with the phytochemicals used alone (Zhou et al., 2016).

Resveratrol is a polyphenol that is mostly found on the skin of red grapes, and sometimes in peanuts and berries. It has antioxidative, anti-inflammatory, anticancer, and cardioprotective properties and has been used as a nutrient supplement. Recently, there are studies of using resveratrol in treating liver fibrosis. Resveratrol could significantly restore levels of liver function biomarkers of oxidative damage, suggesting that the antifibrotic effect may come from the reduced oxidation and HSC inactivation by down-regulating *α*-SMA (Ahmad and Ahmad, 2014). It was also suggested that the antifibrotic effect of resveratrol is because of the inhibition of NF-*κ*B activation, PI3K/Akt phosphorylation, and TLR4 level, which results in attenuation of HSC activation (Zhang et al., 2016).

Deoxyschizandrin is a lignan isolated from the *Schisandra chinensis* (Turcz.) Baill. or magnolia-vine as common name. The fruit of this plant could be prepared into a Chinese medicine five-flavor-fruit, or *Wuweizi*. *Schisandra chinensis* is one of the crucial components in the Chinese prescription, *Fuzheng Huayu Recipe*, to treat liver diseases mainly hepatitis B induced fibrosis (Liu et al., 2019a). Deoxyschizandrin was identified as a compound possessing the function to change the cytochrome P450 enzyme activity during hepatic fibrogenesis (Liu et al., 2019b). It was reported that the metabolism of deoxyschizandrin involved the P450 isoforms CYP3A4 and CYP3A5 (Yang et al., 2015). Deoxyschizandrin was also reported to have liver protective activity by activation of autophagy flux and reduction of apoptosis (Lu et al., 2014).

Ligustrazine, or tetramethylpyrazine, is the bioactive compound isolated from the Chinese herb *Conioselinum anthriscoides* 'Chuanxiong' (Chuanxiong). The antifibrotic action of ligustrazine was reported to be suppression of HSCs *via* SMRT-mediated transrepression of HIF-1*α*. It was shown to attenuate the HSC activities by inhibition of proangiogenic cytokines, suppression of migration and adhesion, restriction of contraction, and lowering the pericyte functions of HSCs (Zhang et al., 2018). The suppression of angiogenic properties of HSCs was also found to be related to the inhibition of canonical hedgehog signaling. SMO, HSP90, and HIF-1*α* were down-regulated while the VEGF and angiopoietin 1 level were depressed by ligustrazine in rat and mice models (Zhang et al., 2017).

Astragalosides are a type of compounds found from the herb *Astragalus mongholicus* Bunge, which are the sources of the Chinese medicine *Huangqi*. Astragalosides are reported to have therapeutic effects on hepatic fibrosis. Its antioxidant properties inhibit the activation of HSCs and regulate MMP-2, TIMP-2, and collagens. The pharmacological pathway was suggested to be TGF*β*/SMAD signaling (Yuan et al., 2018). Astragaloside I, a compound of the astragaloside family, was shown to proliferate inhibition on the HSC cell line LX-2 (Guo et al., 2018). Astragaloside IV, on the other hand, was reported to have an effect on the PI3K/Akt/mTOR signaling pathway in a rat model. It could suppress the inflammatory response so as to limit the fibrogenesis through the action on HSCs (Wei et al., 2019). There were some other reports suggesting the effects of astragalosides on different pathways, including PAR2 and Notch signaling (Mu et al., 2015; Wang et al., 2017).

Paeonol is a phenolic compound isolated from the flowering plant *Paeonia lactiflora* Pall. which roots could be prepared into the Chinese medicine *Baishao*. It was also reported to possess antifibrotic effect as a compound. In a PDGF-induced fibrosis rat model, *Baishao* extract, including paeonol, was shown to inhibit HSC migration and collagen production. The effect was believed to be associated with ERK, p38, and JNK deactivation (Kuo et al., 2012). Paeonol showed similar inhibition effect on HSCs in another CCI_4_-induced fibrosis mice model. Liver injury and fibrosis were decreased through the effect on the TGF*β*/SMAD signaling (Wu et al., 2019b). Paeonol also exhibited its antifibrogenesis effect through the NF-*κ*B inhibition pathway (Kong et al., 2013).

## Network Pharmacology-Associated Study

In order to identify the most important molecular target or pathway of these herbal compounds, we carried out network pharmacology study to understand the global regulation of natural compounds in the treatment of liver fibrosis targeting HSCs. The reported candidate targets of those compounds isolated from the herbal medicine are collected from the Pubmed database. The molecular interactions between the compounds and the targets are analyzed and summarized by the Cytoscape software. The network analysis was performed using “NetworkAnalyzer”, a plug-in function of Cytoscape. The correlation degree of the compounds as well as the gene target was calculated and visualized using the network diagram.

**Figure 3:**
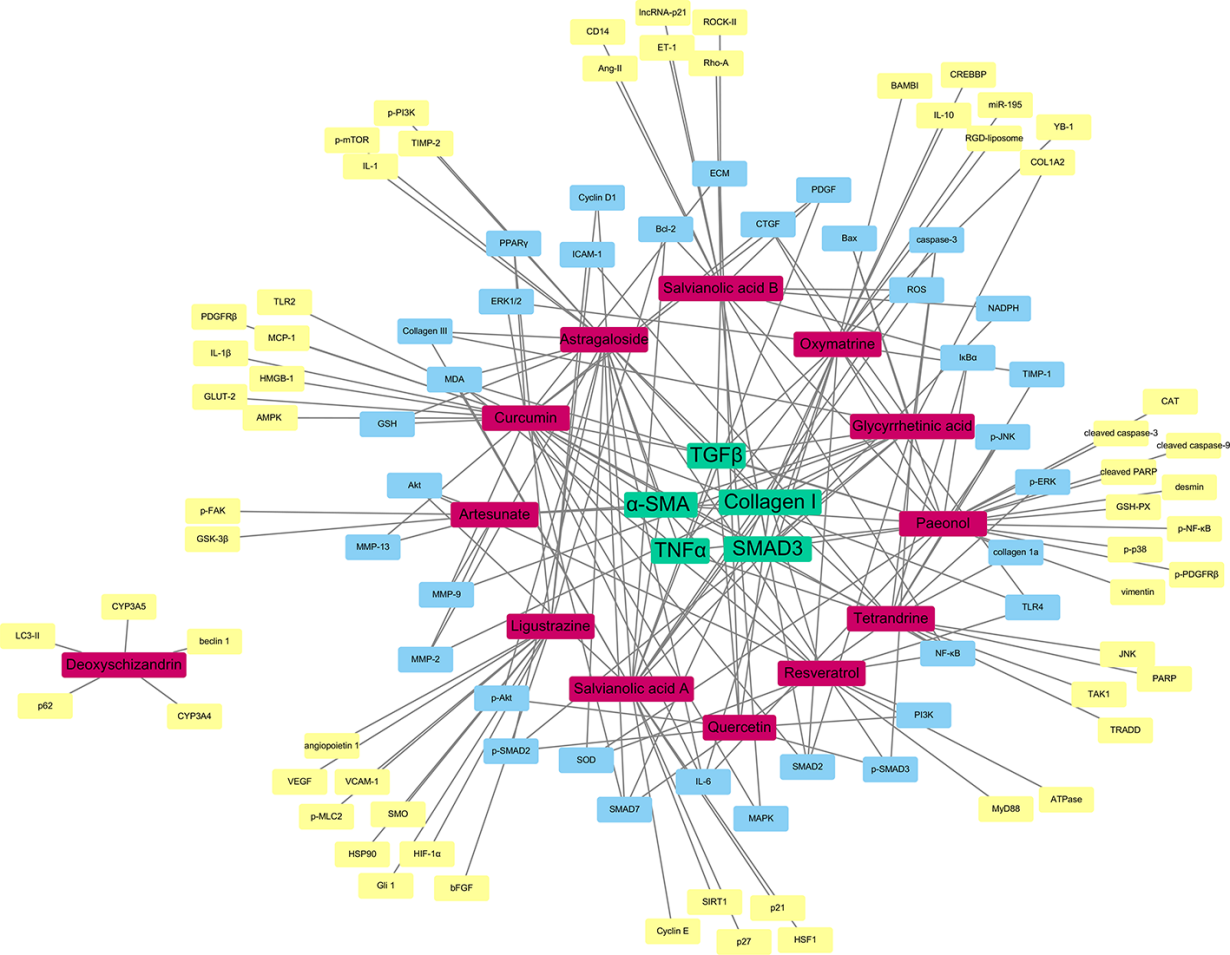
Network pharmacology-based target identification of herbal medicine isolated compounds for the treatment of hepatic fibrosis. TGF*β*, *α*-SMA, Collagen I, TNFα and SMAD3 are the five targets with the highest degree (green). Pure compounds from herbal drugs are labeled in (red). Common targets of the compounds are labeled in (blue), while unique targets of compounds are labeled in (yellow).

As shown as a result in **Figure 3**, the compounds labeled in red color, while the molecular targets are put in the outside region. The genes as common targets to more than one compound are labeled in blue, while the unique targets are labeled in yellow at the outermost area. As a result, the five targets with the highest degree are identified as TGF*β*, *α*-SMA, collagen I, TNFα, and SMAD3 and are located at the center of the network from which, TGF*β* is the most common one and is reported to have interactions with almost all the drugs mentioned in this review.

It is obvious to see that the TGFβ/SMAD signaling pathway play an essential role in the therapy of liver fibrosis through HSCs. The compounds have the aim to lower the TGFβ level of the HSCs, which could slow down the signaling of the particular pathway, and so the expression of *α*-SMA, as the translational product of the pathway and the characteristic of activated HSCs. Moreover, as discussed before, collagen I is an essential indicator of the HSC activation. It is obvious that the therapeutic compounds would have effects on the expression of collagen. The relationship with TNFα signifies the anti-inflammatory nature of the herbal compounds, which is also an important property of antifibrotic drugs. This network pharmacology result shows that, even though there are different herbal medicines reported to have antifibrosis functions, with different active compounds and receptors, the ultimate therapeutic targets may actually be similar, as the most potent curative effect.

## Discussion

Liver fibrosis is becoming a huge public health burden in recent years, due to the unhealthy lifestyles in more developed countries. Progressive fibrosis leading to cirrhosis or eventually cancer will become irreversible, so diagnosis and treatment must be centered in the early stage. Although there are clinical trials for the development of antifibrosis drug, there still lacks a direct therapy method to cure liver fibrosis. Current therapeutic directions include anti-inflammation, antioxidative, and inhibition of effector cells, or HSCs. There are now RAS inhibitors, collagen synthesis inhibitors, antioxidants, PPAR*γ* agonists, and direct inhibition inducers such as IFN*γ* going through different phases of clinical trials. As an ancient wisdom developed for thousands of years, Chinese medicine, or other alternative medicines have their own methodologies in treating liver diseases including fibrosis. Therefore, it is useful to provide insight of the herbal drugs used to treat fibrosis, and there are possibilities to isolate one or more feasible therapeutic compounds.

With the extensive research mentioned previously, it is optimistic that there is indeed more than one compound isolated from herbal medicine that is useful or beneficial to the treatment of liver fibrosis. Many of them are having drug targets on the HSC activators that could help remove the etiology. Since there are limited therapeutic approaches currently, we must extend the research directions to different possibilities, including Chinese herbal medicine. From the result of the network pharmacology, we could clearly see that the herbal compounds are having shared drug targets with each other, and they are consistent with the modern pharmaceutical research also. This greatly raises our confidence on the studies and usage of herbal medicine on the treatment of hepatic fibrosis.

There are some arising clinical trials using Chinese medicine formula as intervention or treatment to target liver fibrosis. For example, the effect of curcumin on diabetes-caused fatty liver diseases was investigated in a randomized placebo-controlled clinical trial (NCT02908152). The TCM Fuzheng Huayu formula was tested in several clinical trials for its antifibrotic activity in viral-induced hepatic fibrosis (NCT00854087, NCT00540397, NCT00543426). The TCM was prescribed to a group of patients while placebo was given as control. The corresponding liver function parameters were measured within different timepoints. However, none of them were shown with promising results. Another trial is using the Fuzheng Huayu tablet with the current approved antiviral drug Entecavir and is currently in the phase 4 clinical trial (NCT02241590). A pilot study using *Yo Jyo Hen Shi Ko*, an herbal-based compound to treat nonalcohol steatohepatitis was performed in a randomized, double-blinded placebo-controlled manner (Chande et al., 2006). All eight patients in the treatment group had a significant decrease in ALT level during the fourth and eighth weeks of intervention, and the level was returned to normal after removing the treatment. Only pilot clinical studies are in progress, and there is still a long way to get an herbal compound approved by the FDA for therapies of liver fibrosis.

There are also some challenges encountered that must be overcome before the extensive clinical use of herbal medicine on the treatment of hepatic fibrosis. One is the possible toxicity issue of the herbs in patients with liver diseases. There are still very limited researches or randomized clinical trial data on the testing of Chinese medicine, especially pure compounds isolated from herbs, and make the progression of the application difficult. Due to the complex nature of the action of herbal medicine on the human body, it is not easy to assess the risk-to-benefit ratio before novel drug development. The pharmaceutical industries are reluctant to carry out related research before extensive scientific evidence is reported. The patients’ benefits are also of a great concern. However, we are positive that these problems and obstacles could be overcome in the coming future and better therapeutic approaches will be developed.

In conclusion, as discussed in the context of the present review, there are some examples of novel bioactive compounds isolated or identified from natural plants or herbal medicines, such as salvianolic acids, oxymatrine, curcumin, tetrandrine, and more, possessing different levels of antifibrosis activity as shown mainly in animal experiments. The TGF*β*/SMAD signaling pathway was identified as the most common drug targets for the compounds *via* network pharmacological strategy. There is sensational possibility of passing through one of them into human clinical trials for the development of derivative drugs. Before that, the exact underlying molecular mechanism must be systematically identified, and the best and useful chemical properties would be kept while improving it with modern derivatives and adjuvants if necessary. This will be foreseeable as a perfect example of the integration of traditional Chinese and modern Western medicine.

## Author Contributions

YF and NW designed the study and prepared the manuscript. Y-TC retrieved data and drafted the manuscript. HT and SL revised and comments the manuscript. All authors confirmed the final manuscript.

## Funding

This study was supported by the Research Grant Council, the HKSAR (Project code: RGC GRF 17152116, 17121419), the Commissioner for Innovation Technology, the HKSAR (Project code: ITS/091/16FX), and the Health and Medical Research Fund (HMRF) (Project code: 16172751).

## Conflict of Interest

The authors declare that the research was conducted in the absence of any commercial or financial relationships that could be construed as a potential conflict of interest.
